# Spontaneous Regression of Brown Tumor in a Patient Treated With Peritoneal Dialysis

**DOI:** 10.7759/cureus.17078

**Published:** 2021-08-11

**Authors:** Kan Ito, Kunihiro Ikuta, Yoshihiro Nishida, Tomohisa Sakai, Shiro Imagama

**Affiliations:** 1 Orthopedic Surgery, Nagoya University Graduate School of Medicine, Nagoya, JPN; 2 Rehabilitation, Nagoya University Hospital, Nagoya, JPN

**Keywords:** brown tumor, giant cell tumor of bone, hyperparathyroidism, radius, peritoneal dialysis

## Abstract

A 52-year-old man, with a history of diabetic nephropathy and renal cancer, had been treated with peritoneal dialysis for four months before consulting our hospital. At the time of imaging evaluation, three years after surgery for renal cancer, fluorodeoxyglucose accumulation was found at the distal metaphysis of the left radius. After the biopsy, he was diagnosed with giant cell tumor of bone (GCTB), and surgery was scheduled. However, osteogenesis was observed in the images retaken before surgery. It was found that his intact parathyroid hormone level had been abnormally high four months prior to his visit to us but had subsequently normalized. The tissue obtained by re-biopsy revealed osteogenesis with the disappearance of multinucleated giant cells, suggesting a brown tumor (BT). The tumor was thought to have been caused by secondary hyperparathyroidism (HPT) associated with peritoneal dialysis. When osteolytic lesions mimicking GCTB are found, the possibility of BT should be considered based on comorbidities and clinical information.

## Introduction

Brown tumor (BT) is a non-neoplastic lesion that reflects abnormal bone resorption caused by hyperparathyroidism (HPT) [1]. It can be either monostotic or polyostotic and affects any bone in the body, most commonly facial bones, clavicle, ribs, pelvis, and femur [2]. However, due to the similar pathological findings with multinucleated giant cells and osteolytic imaging findings, not a few cases are misdiagnosed as giant cell tumor of bone (GCTB). Here, we present an unusual case of a bone lesion diagnosed as GCTB in a patient with chronic kidney disease (CKD) whose diagnosis was later revised to BT.

## Case presentation

A 52-year-old man was referred to our hospital because of abnormal accumulation at the distal metaphysis of the left radius on an 18F-fluorodeoxyglucose-position emission tomography (FDG-PET) scan taken for follow-up after renal cancer. The patient had a history of CKD due to microvascular nephrotic syndrome and diabetic nephropathy. The patient had undergone a unilateral partial nephrectomy for renal cancer three years before and had not experienced any recurrence or metastasis.

At the time of the initial examination, there was no obvious swelling or tenderness in the distal part of his left forearm and no restriction in the range of wrist motion. Laboratory data showed slightly low levels of various electrolytes in addition to renal dysfunction, consistent with the hemodialysis state, with no other noteworthy findings: Na 134mEq/L, K 3.2mEq/L, Cl 92mEq/L, Ca 7.4mg/dL, BUN 75.0mg/dL, Cr 10.01mg/dL.

**Figure 1:**
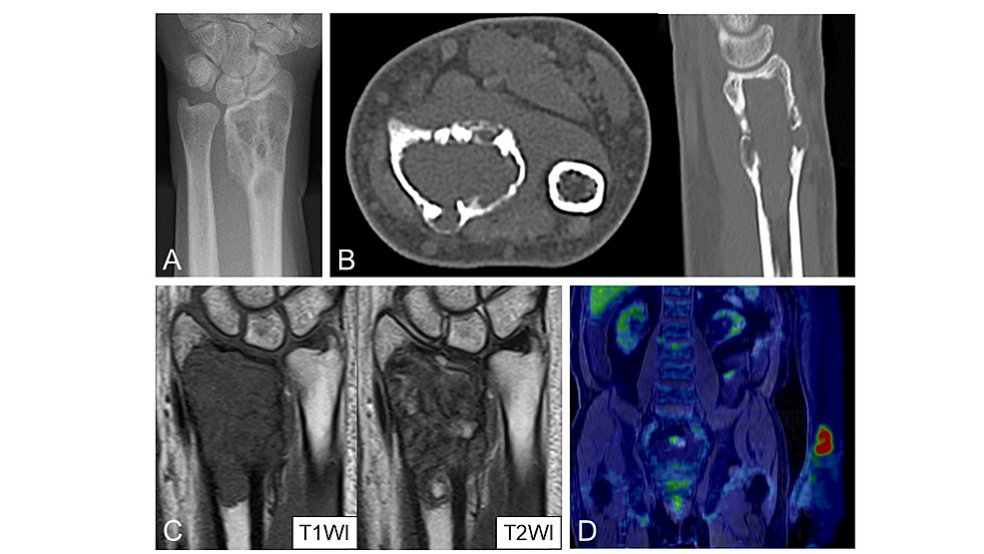
Images at the time of referral to our hospital. A: X-ray image showed a geographic radiolucent lesion with sclerotic margins. B: CT image depicted no calcification inside the tumor, and the bone cortex appeared to be partially disrupted. C: MRI images showed homogeneous isointense signals in the T1-weighted images and heterogeneously mixed iso- and high-signals in the T2-weighted images. D: FDG-PET image revealed high accumulation at the left distal radial metaphysis.

A geographic radiolucent lesion with sclerotic margins was found in the radiographs taken at the initial consultation (Figure 1A). The tumor measured about 5 cm, and the bone cortex was thinned and expanded due to tumor growth. Computed tomography (CT) images showed no calcification inside the tumor, and the bone cortex appeared to be partially disrupted (Figure 1B). Magnetic resonance imaging (MRI) showed homogeneous isointense signals in the T1-weighted images and heterogeneously mixed iso- and high-signals in the T2-weighted images (Figure 1C). The FDG-PET scan revealed high accumulation in the left distal radial metaphysis (Figure 1D).

**Figure 2:**
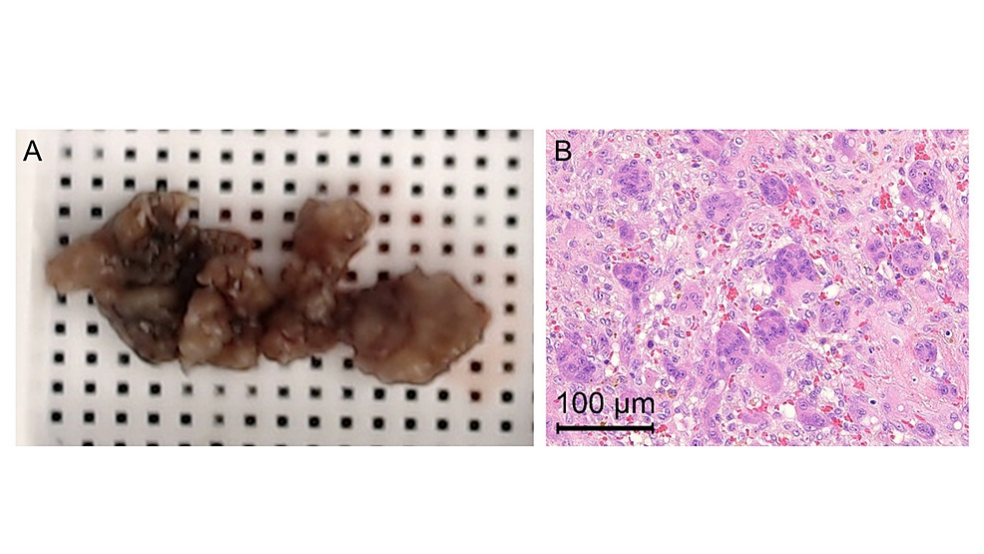
Samples obtained at the time of biopsy and their pathological findings A: The biopsy samples were brownish in color. B: The pathological findings showed a number of multinucleated giant cells and mononuclear neoplastic cells with proliferation of capillaries.

One month after the initial visit, an incisional biopsy of the tumor was performed with the possibility of a low-grade malignancy based on the imaging findings. Tissue samples were collected by making a hole on the bone cortex just subjacent to the first compartment of the extensor tendon sheath. After the biopsy, the bone hole was filled with cement, the tendon sheath was repaired, and the procedure was completed. The collected samples were brownish (Figure 2A). The pathological findings of the tissue revealed numerous multinucleated giant cells and no atypical cells, leading to the diagnosis of GCTB (Figure 2B).

**Figure 3:**
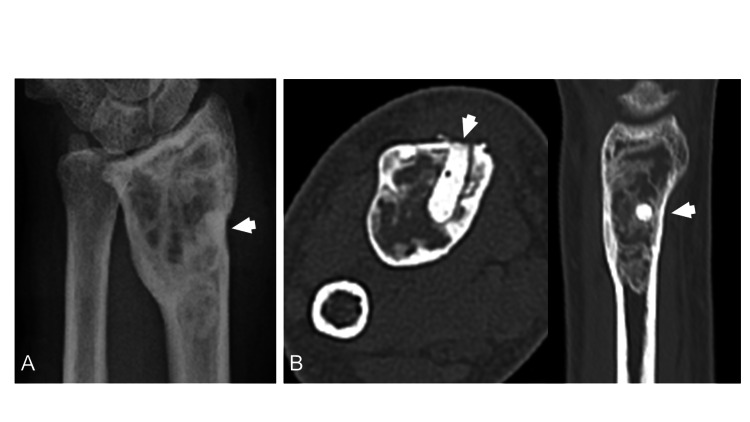
X-rays and CT images taken again before surgery Both X-ray image (A) and CT images (B) showed osteogenesis findings. (Arrows indicated the cement used to fill in the biopsy area.)

The patient was scheduled to undergo tumor curettage. Administration of denosumab may be a treatment option, but was not indicated because of the frequent hypocalcemia at different times caused by CKD. However, X-rays and CT images taken again before surgery showed osteogenic signs in the tumor (Figure 3). Usually, GCTB do not show osteogenesis in their natural course. Therefore, it was necessary to reconsider the diagnosis of the biopsy specimen.

Reviewing the laboratory data, the intact parathyroid hormone (PTH) level was abnormally high (915.4 pg/mL) at the time of the initiation of peritoneal dialysis four months before the initial visit. Thereafter, the value of intact PTH decreased to within the normal range (20.0 pg/mL). These laboratory data are shown in Table 1. These data suggested a possible association between PTH and tumor behavior.

**Table 1:** Transition of blood laboratory data four months before and after the initial visit to our department. Na - sodium; K - potassium; Cl - chlorine; Ca - calcium; P - phosphorus; BUN - blood urea nitrogen; Cr - creatinine; PTH - parathyroid hormone.

	Normal range	Four months before the initial visit	Four months after the initial visit
Na	135-145mEq/L	134 mEq/L	132 mEq/L
K	3.5-5.0 mEq/L	4.0 mEq/L	3.7 mEq/L
Cl	98-108 mEq/L	98 mEq/L	91 mEq/L
Ca	8.6-10.2 mg/dL	9.7 mg/dL	8.9 mg/dL
P	2.5-4.5 mg/dL	5.5 mg/dL	6.3 mg/dL
BUN	8.0-20.0 mg/dL	69.5 mg/dL	62.5 mg/dL
Cr	Male:0.65-1.09mg/dL	8.28 mg/dL	12.69 mg/dL
Intact PTH	10-65 pg/mL	915.4 pg/mL	20.0 pg/mL

**Figure 4:**
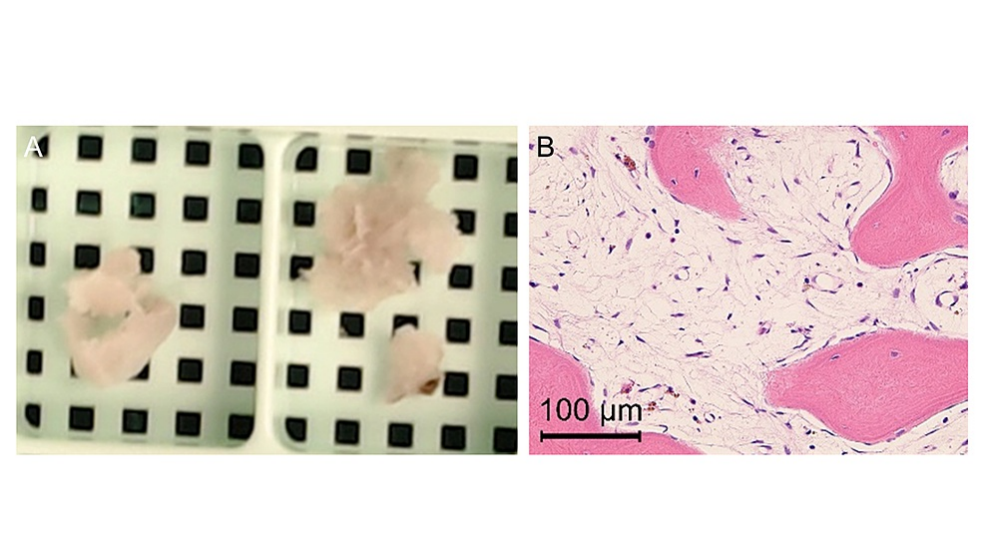
Samples and pathology findings at re-biopsy. A: The re-biopsy samples were white tissue with a light pinkish color. B: The pathological findings showed the mature bone tissue and the almost complete disappearance of multinucleated giant cells.

**Figure 5:**
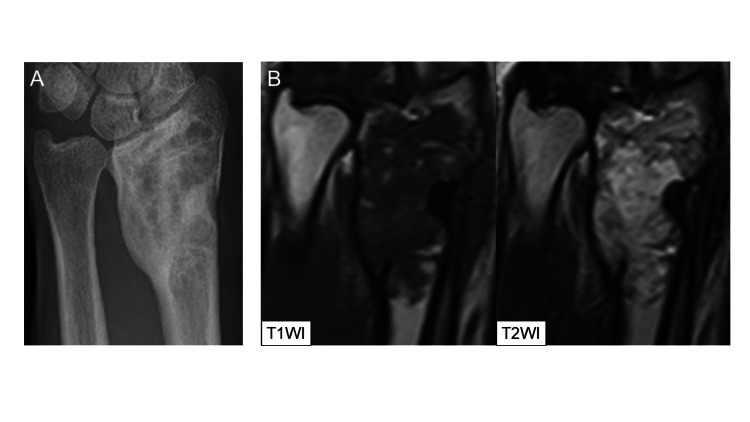
Images at one year after biopsy. A: The X-ray image showed further osteogenic features. B: The MRI images showed that the tumor borders became unclear and the tumor turned to a higher intensity on T2-weighted images.

We decided to perform a re-biopsy instead of curettage in order to confirm the pathological diagnosis. The newly collected samples were white tissue with a light pinkish color (Figure 4A), differing from those of the previous biopsy. The pathological findings of re-biopsy specimens showed the almost complete disappearance of multinucleated giant cells and mature bone tissue, with no obvious neoplastic changes or malignant images (Figure 4B). The osteogenic changes and the disappearance of the giant cells indicated that the tumor was a BT. The patient was followed up by clinical images every six months. One year after the biopsy, the radiographic images showed further osteogenesis and the lesion was considered almost healed (Figure 5).

## Discussion

GCTB is an osteolytic neoplasm that usually develops in the metaphysis to epiphysis of a long bone or the axial skeleton and occurs commonly in 20-40-year-olds [3,4]. It is classified as an intermediate tumor type because of its local destructive potential and metastatic potential. Histologically, conventional GCTB is composed mainly of three types of cells: mononuclear neoplastic cells, mononuclear histiocytic cells, and osteoclast-like multinucleated giant cells [5]. The primary treatment is curettage or excision of the lesion [6]. In cases that surgical treatment is complex, denosumab administration can be used for treatment [7]. Denosumab may also be given preoperatively to reduce the risk of bleeding during GCTB surgery [8].

BT is a non-neoplastic osteolytic reactive lesion that reflects abnormal bone resorption caused by HPT [1]. There is dark, reddish-brown coloration induced by intralesional hemorrhage and hemosiderin deposition that gives the lesion its name [9]. In addition, we consider this case report to be a rare and valuable one for which there are photographs of the actual tissue collected that document the changes in color tone. BT also has multinucleated giant cells histologically, and there have been some reports of its misdiagnosis due to similarities in the imaging and pathological findings with GCTB [10]. BT does not require surgical resection; instead, it regresses with the treatment of HPT [11].

HPT, which causes hypercalcemia, can be classified into primary and secondary, with most primary cases resulting from a single parathyroid adenoma [12]. Secondary HPT is caused by prolonged renal insufficiency and results in hypocalcemia [13]. In this case, it is assumed that the patient had secondary HPT associated with CKD. The introduction of peritoneal dialysis and electrolyte correction with phosphorus adsorbent and vitamin D medication could control the PTH levels and promoted regression of the lesion. Most of the previous literature described that a BT developed as a result of primary HPT or secondary HPT due to hemodialysis. Our report is unusual in that the tumor regressed after medical treatment, including peritoneal dialysis, for a BT that developed in a patient with severe CKD.

It is essential to check for comorbidities such as CKD and HPT in patients diagnosed with GCTB. In those patients, several reports recommend that serum calcium and PTH levels be measured to rule out the possibility of BT [14]. From the point of view of pathological differentiation, multinucleated giant cells may form clusters in BT, but they are often distributed diffusely in GCTB [15]. Furthermore, GCTB is reported to have a point mutation at H3F3A in 96% of cases [16]. About 90% of the mutations are p.G34W mutations, and p.G34L, p.G34M, p.G34R, or G34V mutations are also present in fewer than 2% each [17]. Recent reports indicate that immunostaining with H3.3 G34W is helpful for differentiating GCTB from similar diseases with multinucleated giant cells [18-20].

## Conclusions

In this case, even after the initial diagnosis of GCTB, this had to be reconsidered because the clinical course was so different from that of a typical GCTB. BT presented similar imaging and pathological findings to those of GCTB. When the pathological diagnosis was GCTB, it was necessary to investigate the possibility of BT based on the presence of comorbidities and various clinical information. When BT cannot be ruled out, it is useful to evaluate blood tests for calcium, phosphorus, intact PTH, and other pertinent clinical parameters.
